# Introducing effective parameters for predicting job burnout using a self-organizing method based on group method of data handling neural network

**DOI:** 10.1371/journal.pone.0290267

**Published:** 2023-11-06

**Authors:** Tingting Fan, Ehsan Nazemi

**Affiliations:** 1 Assumption University, Bangkok, Thailand; 2 Faculty of Engineering and Physical Sciences, Southampton University, Southampton, United Kingdom; Houston Methodist Academic Institute, UNITED STATES

## Abstract

In addition to affecting people’s bodily and mental health, the Covid-19 epidemic has also altered the emotional and mental well-being of many workers. Especially in the realm of institutions and privately held enterprises, which encountered a plethora of constraints due to the peculiar circumstances of the epidemic. It was thus anticipated that the present study would use a group method of data handling (GMDH) neural network for analyzing the relationship of demographic factors, Coronavirus, resilience, and the burnout in startups. The test methodology was quantitative. The research examined 384 startup directors and representatives, which is a sizable proportion of the limitless community. The BRCS, the MBI-GS, and custom-made assessments of stress due to the Coronavirus were all used to collect data. Cronbach’s alpha confirmed the polls’ dependability, and an expert panel confirmed the surveys’ authenticity. The GMDH neural network’s inherent potential for self-organization was used to choose the most useful properties automatically. The trained network has a three-layered topology with 4, 3, and 2 neurons in each of the hidden layers. The GMDH network has significantly reduced the computational load by using just 7 parameters of marital status, stress of covid-19, job experience, professional efficiency, gender, age, and resilience for burnout categorization. After comparing the neural network’s output with the acquired data, it was determined that the constructed network accurately classified all of the information. Among the achievements of this research, high accuracy in predicting job burnout, checking the performance of neural network in determining job burnout and introducing effective characteristics in determination of this parameter can be mentioned.

## 1. Introduction

On March 11, 2020, the World Health Organization officially labeled the widespread spread of Coronavirus as a pandemic. The main incident was explained in Wuhan, China [1]. From the beginning of this pandemic through September 30, 2021, insights predict a total of 233,136,147 infected cases worldwide, with 4,771,408 fatalities [2]. Coronavirus isn’t just a major health emergency, though; the virus is also altering the global demand structure, with consequential consequences for the economy and business [3]. In addition to weakening people physically, such pandemics will have an impact on their mental health, too [4]. Many residents’ joy has been dampened as a result of the coronavirus, although the disease’s effects vary from person to person depending on a host of demographic and societal variables [5]. Residents were required by quarantine rules to limit their social interactions during the pandemic in an effort to prevent the spread of disease [6]. Restrictions on things like work stoppages, entertainment shutdowns, extended hours at emergency clinics and clinical centers, travel bans, and so on were upheld in many countries. Some people experienced anxiety and mental distress as a result of these norms and constraints, as well as the fears and tensions brought on by the spread of the Coronavirus within communities, the resulting decrease in correspondence and collaboration, and the subsequent drop in business profits [7]. Thus, burnout is one of the most consequential outcomes of the chronic stress caused by the Coronavirus; burnout is a mental condition characterized by distressing contact with others, more responsibilities, worse job quality, and a lack of social involvement [8]. When this happens, a person’s job stops being as significant in their life. A person who has hit burnout is one who is continually exhausted, has a dominant mental attitude, is quite cynical and doubtful in interpersonal interactions, and is generally pessimistic [9]. For this reason, a number of medical professionals have pondered the significance of unique skill sets that enable individuals to endure adverse situations [10], Considering that it is generally accepted that people have negative effects on their mental health as a direct result of exposure to a variety of crises and confrontations. The medical community is in agreement that some individuals respond differently to trauma because of underlying psychiatric illnesses [11]. Thus, researchers endeavor to cultivate internal factors that enhance human resilience and health [12]. A person’s level of resilience could increase or alter throughout time. Being resilient in the face of adversity requires the capacity to rapidly recover from setbacks. As a result, resilience has come to represent successful adjusting to novel circumstances and overcoming setbacks [13]. Ability to overcome problems and grow as a result is what "resilience" [14] refers to. The World Health Organization confirmed 5,587,040 cases of Coronavirus as of September 30, 2021, with 120,428 deaths attributable to the virus [15]. Director and employee burnout are inevitable in light of the past restrictions and closures of numerous organizations, including startups, for controlling Coronavirus. This is because a great percentage of these businesses have had to cut down on staff, activities, offers, and revenue, or perhaps completely shut down. Coronavirus has been around for about three years, and it has already imposed limitations on small businesses, which, in contrast to larger ones, cannot survive in insecure circumstances for very long owing to their lack of resources and young age. Since there is no easy way to reduce the pressure on these startups’ leaders, the continual stress has led to a vulnerable state that need for constant attention. The investigation of the factors that led to burnout among startup managers and staff during the Coronavirus pandemic, as well as an assessment of the degree to which these factors are related to demographic traits and resilience, were the main objectives of this research.

A person’s wellbeing is impacted by their home’s physical, emotional, and social settings throughout time. It cannot be avoided; the majority of people spend their waking hours at work [16]. Industrial psychologists have developed a greater focus on the causes, symptoms, and effects of burnout in the workplace throughout time. The term "burnout" [17] describes the emotional and physical tiredness, resentment, loneliness, and sadness that may be brought on by a protracted period of work stress, such as that which self-employed people encounter. This issue has had negative effects on businesses, institutions, and employees [18]. The stresses of the workplace and an incompatibility between an individual’s character attributes and the requirements of their jobs are two primary causes of burnout [19]. For some workers, the stresses of the job might become too much to bear, leading them to rely on ineffective and inefficient coping mechanisms, eventually leading to burnout [20].

The notion of resilience has been examined and studied in many different scientific domains, including supply chains [21, 22], crisis medicine [23], management [24], and cooperative networks [25], but now, a dedicated concept has not been considered for it. Francis and Bekra (2014) describe resilience as the capacity of a system to lessen the impact of shocks, absorb them if they do occur, and swiftly regain normal operation afterward [26]. According to Newman (2005), resilience is defined as "the capacity to deal effectively with adversity" [27]. Stress management strategies that draw on resilience principles have been shown to be effective [28]. Recently, there has been a lot of focus on resilience, which may be defined as the study and discovery of one’s own strengths and insights. Progress and resistance are the results of resilience under trying circumstances [29]. In [30] researchers tried to predict burnout using radial basis function (RBF) neural network, but the failure to select important characteristics caused a large number of characteristics to be applied as input.

The main objectives of the current research are as follows: First, managers and workers at new businesses are studied to learn more about the connection between resilience and burnout. As a second objective, we look at how covid-19 stress affects the resiliency of startup employees and managers. Third, we investigated the function of GMDH neural network in predicting job burnout. Fourth, the effective characteristics on job burnout were introduced using the self-organization capability of GMDH neural network

## 2. Materials and methods

### 2.1 Design and procedure

This study makes use of the rationale in a quantitative cross-sectional design. Entrepreneurial leaders and their representatives made up the review pool. A sample of 384 people was selected in accordance with standards for estimating sample sizes from infinitely many populations. The samples were collected in the first half of 2021 (August–September). The information utilized in this study was gathered from previous research [31]. The methods of sampling and data collection are given in detail in the mentioned article. The researchers in this study employed a questionnaire to compile their findings.

#### 2.1.1 Maslach Burnout Inventory ‐ general survey (MBI-GS) [32, 33]

The three components of this shape are fatigue, competence in one’s chosen career, and cynicism. There are six components to exhaustion, six to professional effectiveness, and five to cynicism. Excessive skepticism decreased productivity at work, and overwhelming fatigue are all symptoms of burnout. A Likert scale with a 1 to 5 ratings range could be used to evaluate your answers to each of the 4 questions on this form. On a 5-point scale, the responses run through the range from "this item does not reflect me at all" = 1 to "this item reflects me completely" = 5. The apparatus’s validity and reliability are confirmed by the study’s use of the gold-standard questionnaire. With the 22-item instrument, for the fatigue dimension, the Cronbach’s alpha was 0.9, for cynicism it was 0.79, for professional efficiency it was 0.71, and for all things combined, it was 0.76. Cronbach’s alpha for this research was 0.82, and it was validated via the use of an expert panel and a pilot study.

#### 2.1.2. Brief Resilience Coping Scale (BRCS) [27]

A Likert scale with a 1–5 rating range may be used to assess your answers to each of the four questions on this form. On a 5-point scale, the responses run through the range from "this item does not reflect me at all" (a 1) to "this item represents me entirely" (5). Scores of 17 and above suggest a great degree of resilience and flexibility in this form. Both the expert panel’s evaluation and the pilot test confirm the survey’s validity and reliability (Cronbach’s alpha = 0.86). Cronbach’s alpha was determined to be 0.83 in this instance.

#### 2.1.3 Measurement of Covid-19 stress using a researcher-created questionnaire

In addition to the six questions used to evaluate Covid-19 stress levels, this survey also inquires as to the respondent’s gender, age, employment, marital status, and family size. This form has been found to be valid and reliable after being reviewed by a team of experts, with a Cronbach’s alpha of 0.79. Managers and employees at startups were approached about participating in the study once they had seen and agreed to the protocol. It’s also important to stress that complete confidentiality has been ensured for all information collected in this study.

### 2.2 Participants

Table 1 provides a breakdown, by demographics, of the tested statistical samples. 167 females, 43.5%, and 217 males, 56.5%, create the research sample sizes, which varied from 167 to 217. In addition, men and women have an average job history of 14.63 and 11.86 years, respectively. There were 133 married women and 34 single women among the women who were questioned, and 131 married men and 86 single men among the males. In the data pool, 100 females and 118 males were parents.

**Table 1:** Synopsis of the demographic makeup of the data set [31].

Gender	Average job experience (years)	Number (Person)	Marital Status (Person)		Average age (years)	Children Status (Person)	
			Single	Married		Presence of Child	No Child
Female	11.86	167	34	133	40.43	100	67
Man	14.63	217	86	131	42.84	118	99
Total	13.25	384	120	264	41.64	218	166

## 3. GMDH neural network

Descriptive statistics and inferential statistics were calculated using SPSS. A GMDH neural network is programmed in MATLAB. In this research, MATLAB software was used to implement GMDH neural network. In MATLAB, there are many pre-designed toolboxes for implementing different neural networks, but there is no toolbox for implementing GMDH neural network. For this reason, in this research, we have coded all the steps of training and testing this neural network manually step by step. In this investigation, a neural network was fed ten indicators of burnout on the job to establish its existence or absence. The existence of job burnout is represented by 1 and its absence by 2. The input data consists of seven qualitative factors (number of years in the employment, age, exposure to Covid-19 stress, level of weariness, level of cynicism, level of professional efficiency, and level of resilience) and three nominal variables (married status, gender, and having children).

Artificial neural networks are widely recognized as one of the most trustworthy, insightful, and accurate analytical tools for approximating complicated and non-linear processes. Several scientific fields use neural networks for modelling, prediction, classification, pattern recognition, etc. This includes engineering, medicine, and experimental sciences.

In 1968, M.G. Ivakhnenko created a smart approach to handling complicated and non-linear issues called the Group Method of Data Handling (GMDH) [34]. These algorithms really generate auto techniques that can forecast, classify, synthesize controls, and troubleshoot systems. Ivahnenko’s algorithm automatically identified the number of layers in a network, the weighting of each input data point, and the ideal approach to construct the network. Kolmogorov-Gabor polynomial expresses the relationship between input and output in this neural.


$$y=a_{0}+\sum_{i=1}^{m}a_{i}x_{i}+\sum_{i=1}^{m}\sum_{j=1}^{m}a_{ij}x_{j}x_{j}+\sum_{i=1}^{m}\sum_{j=1}^{m}\sum_{k=1}^{m}a_{ijk}x_{i}x_{j}x_{k}+\cdots$$ (1)

Where y, x (x_1_, x_2_,…, x_m_), and a (a_1_, a_2_, …, a_m_) are represent the output of network, the features vector, and the coefficient or weight, by, respectively. If a GMDH network was to function, the following steps would have been taken:

The first step is to create the new variables $z_{1},z_{2},\cdots ,z_{\left(\frac{m}{2}\right)}$. At this point, we calculate the quadratic regression polynomial for all $\left(\begin{array}{c} m\\ 2 \end{array}\right)$ combinations of independent variables (features) (x_1_, x_2_, …, x_m_) using Eq (2).


$$z=c_{1}+c_{2}x_{i}+c_{3}x_{j}+c_{4}x_{j}^{2}+c_{5}x_{j}^{2}+c_{6}x_{j}x_{j}$$ (2)

In this process, determining the c_i_ coefficients is the goal. These coefficients were computed using the least squares method. The resultant $\left(\begin{array}{c} m\\ 2 \end{array}\right)$combination of quadratic regression polynomials is close to the target function. Second, inactive neurons are those that failed to provide a reliable output prediction. Those neurons that are left are put to work in the layer-up process. In addition to laying down the foundation for subsequent layers, this process also picks out the most effective neurons. In Step 3, we use the polynomial we discovered in Step 2 to create the following layer. This means the previous polynomial is employed as a building block for the next polynomial, and the second procedure is repeated until a good neuron is found. Iterations of this process are carried out several times until the complete GMDH neural network is formed. Because of its progressive and supervised nature, the GMDH network requires three distinct phases of training, validation and testing before it can be put into action. About 70% of the data are chosen at random in the training phase and utilized to create the model. At this point, the framework of the neural network is being established. Validation data, which makes up 15% of the total, is used to put the network to the test while it is being trained. The proper learning method has been used if the network correctly reacts to this data. The remaining 15% of the data are finally applied to the network’s input to assess how well the network performed using the data that it had not previously seen in order to guarantee that the neural network has been constructed correctly. The successful processing of these three data sets by the neural network assures the network’s proper functioning under operating settings.

**Fig 1:**
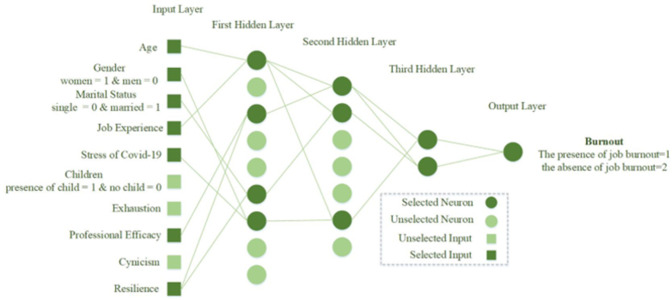
Schematic of the constructed GMDH neural network.

**Fig 2:**
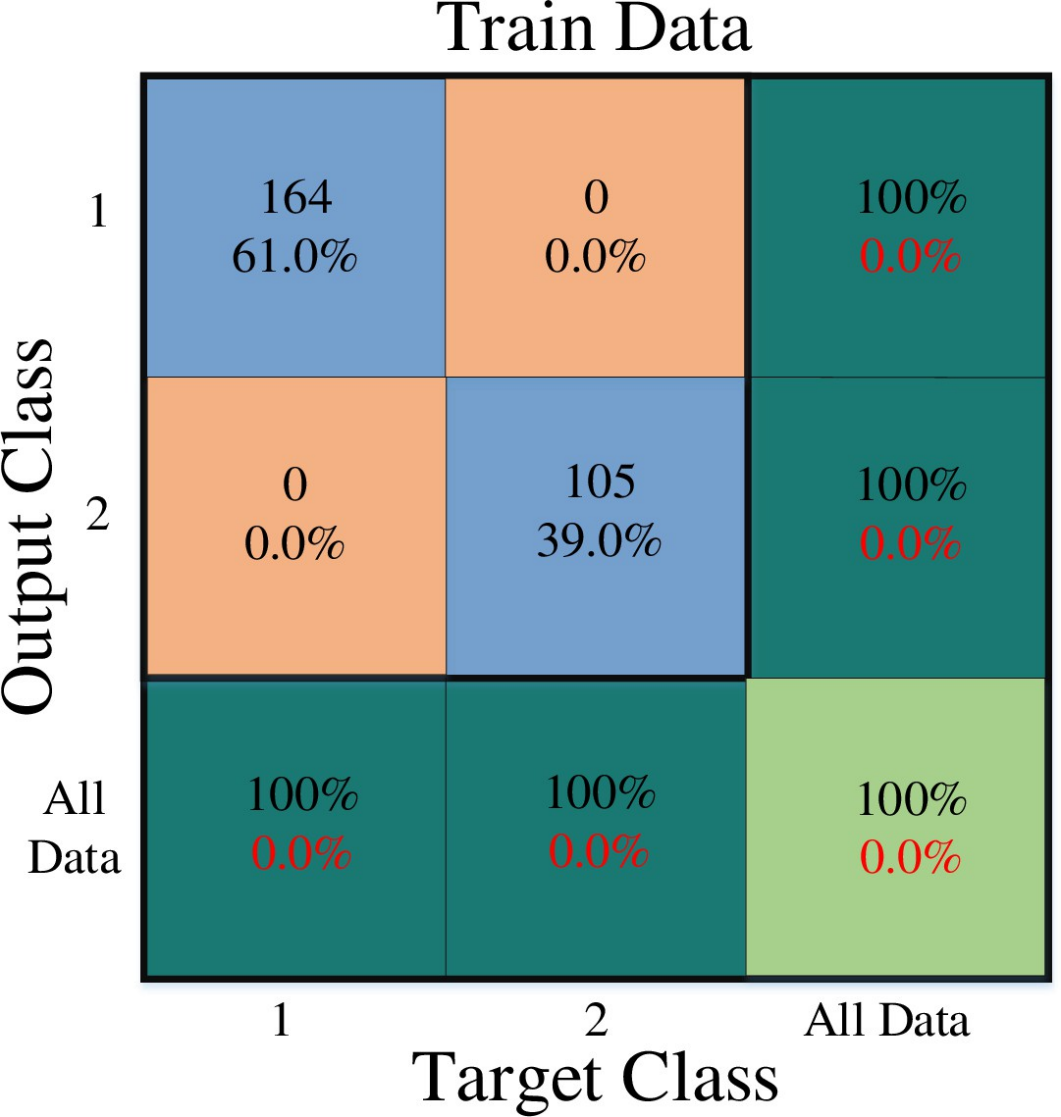
Train data confusion matrix.

**Fig 3:**
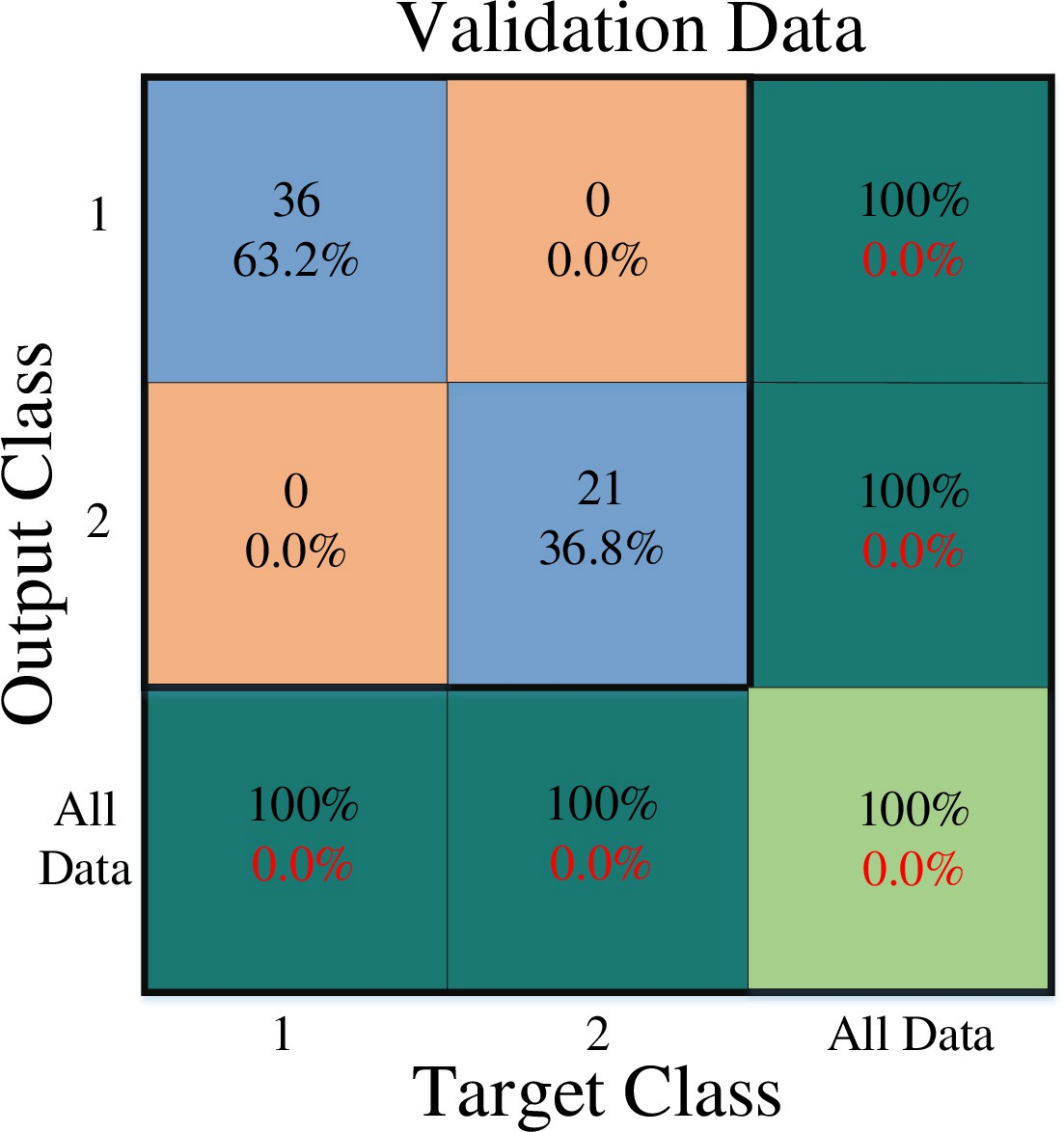
Validation data confusion matrix.

**Fig 4:**
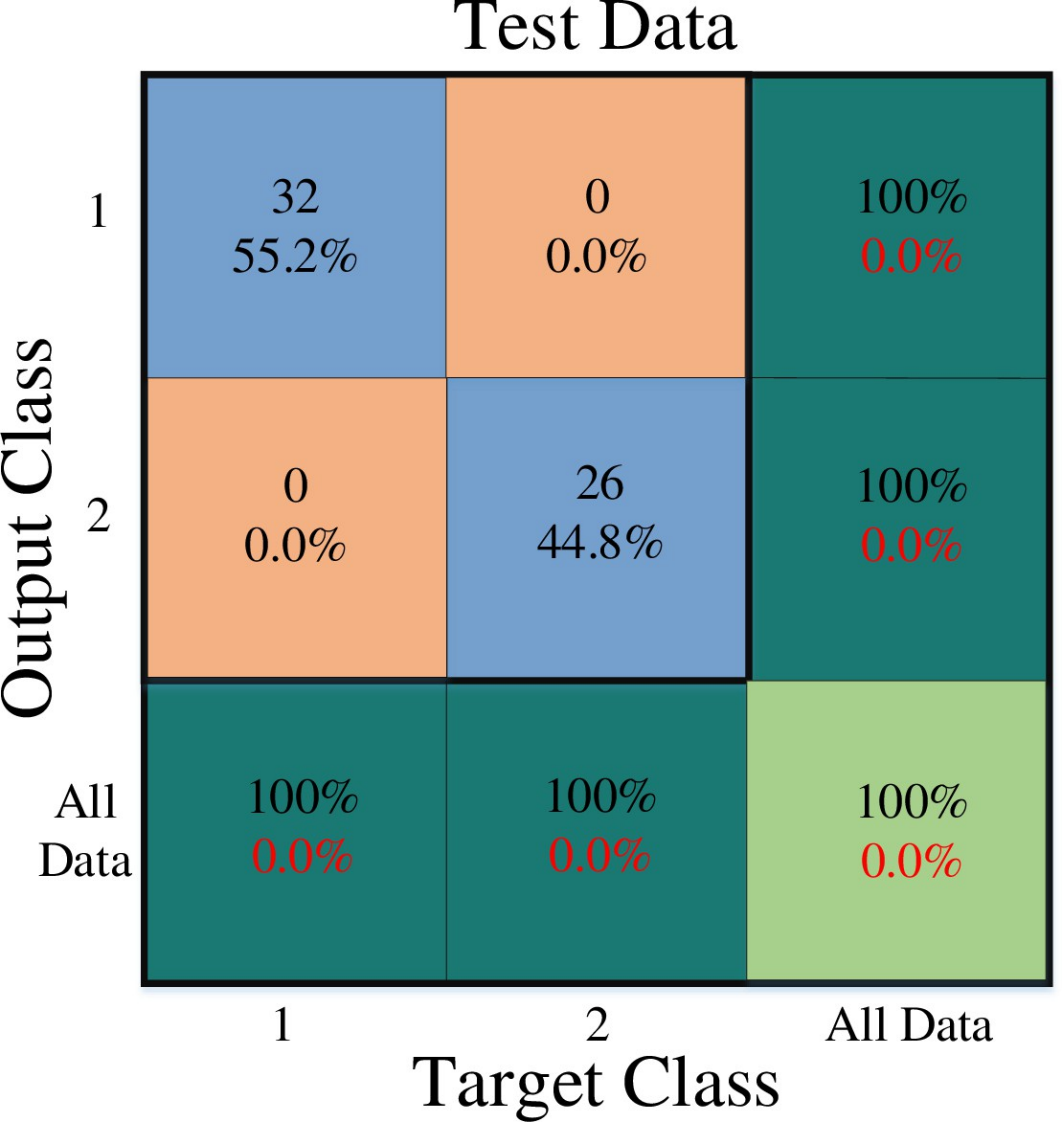
Test data confusion matrix.

Fig 1 depicts the overall network architecture of the created neural network. The number of operational neurons in the network’s three hidden layers are respectively 4, 3, and 2. The GMDH neural network is formed in a self-organized manner. Seven input features are chosen in this arrangement. These were the values marital status, stress of covid-19, job experience, professional efficiency, gender, age, and resilience. Therefore, these factors may be used to predict burnout. At this most profound level, 1 denotes the presence of work burnout while a 2 denotes the absence of it. It is important to say that the first and second classes are divided by a cutoff value of 1.5, with class 1 having data with a value less than or equal to 1.5 and class 2 including values higher than 1.5. All outputs were accurately classified by the specified neural network. In order to demonstrate how well the constructed network performs on training, validation, and test data, the confusion matrix is used are shown in Figs 2–4.

## 4. Results

The findings of the Pearson correlation analysis revealed a direct and significant association between burnout and exhaustion (*rp* = 0.594, *p* < 0.001) and cynicism (*rp* = 0.467, *p* < 0.001), but a negative and significant relationship with professional efficiency (*rp* = -0.322, *p* < 0.01). Additionally, there is a strong inverse association (*rp* = -0.222, *p* < 0.01) between resilience and burnout, indicating that as resilience increases, burnout levels drop. Inferential statistics revealed that the COVID-19 stress rate was 6.24 for males and 8 for women, indicating that women experience more stress than men do. Additionally, married women with children reported 8.94 and married men 7.97 for this stress. According to Pearson analysis, there is a strong negative correlation between COVID-19 stress and resilience, which means that as COVID-19 stress increases, resilience also reduces (*rp* = -0.306, *p* < 0.01). Based on statistical analysis, there was a direct and substantial correlation between COVID-19 stress and burnout, which meant that as COVID-19 stress grew, so did the amount of burnout (*rp* = 0.499, *p* < 0.001). Additionally, the stress of COVID-19 has a direct and significant association with exhaustion (*rp* = 0.532, *p* < 0.001), an inverse and significant relationship with professional effectiveness (*rp* = -0.200, *p* < 0.01), and a direct and significant relationship with cynicism. (*rp* = 0.427, *p* < 0.001). According to the findings addressing the resiliency of startup managers and workers, men, and women exhibit an average resilience of 13.73 compared to 11.86 for women. Additionally, married women with children had a resilience rate of 9.44, while married males with children had a resilience rate of 10.06. The resilience rate was 15.76 for single women and 17.77 for single males at the end. These findings support the notion that, under the resilience questionnaire’s criteria, where a score of 13 or below denotes poor resilience, single persons have a greater degree of resilience than married people. The findings showed that 134 individuals had strong resilience, 194 people had poor resilience (about 35% of the statistical population), 56 people had moderate resilience (around 15% of the statistical population), and 194 people had high resilience (roughly 35% of the statistical population).

Inferential statistics, on the other hand, reveal that 232 out of 384 members of the statistical population (61%) suffer from burnout syndrome, which is characterized by high exhaustion, poor professional effectiveness, and high cynicism. Among them, 115 of them are women and 117 are males. The findings also indicate that married people (221) experience burnout at a greater rate than single people. The exhaustion variable had a mean score of 3.99, the professional efficiency variable had a mean score of 2.97, and the cynicism variable had a mean score of 3.72. These mean scores were higher for the two variables of exhaustion and cynicism than the average for the Likert scale, while the mean score for the professional efficiency variable was lower [31].

**Fig 5:**
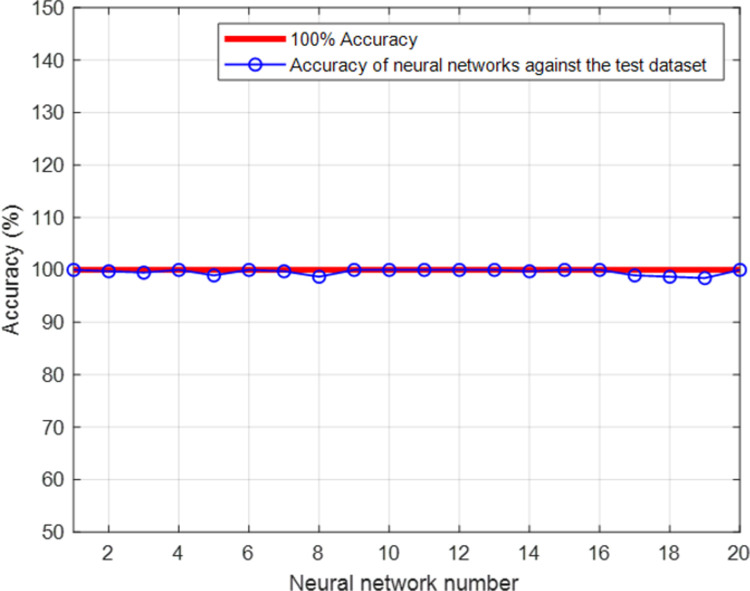
The result of 20-fold cross-validation.

The primary purpose of this study was to use artificial neural network architecture to investigate the connection between demographic factors, resilience, covid-19, and job burnout in the context of startup companies. There is good value in studying how Covid-19’s stress has affected the health of enterprises, the productivity of management, and the well-being of workers. Therefore, ten factors, including age, COVID-19 stress, marital status, gender, years of work experience, children, resilience, professional effectiveness, cynicism and exhaustion, and the extent to which one feels burned out on the job, could be attributed to the very precise GMDH network. To predict job burnout, the developed neural network employed just seven factors of job experience, stress of covid-19, marital status, professional efficiency, age, gender, and resilience. Therefore, the chosen criteria are more crucial in establishing the existence or absence of burnout in the whole of the data obtained. This study’s computation volume is also less than in earlier research since fewer inputs were used. Using a GMDH neural network, this study improves the accuracy of job burnout predictions while decreasing the system’s computational load. The multilayer perceptron (MLP) neural network was used to identify job burnout in a previous study [31], however, there was a significant gap in this study since the neural network was unable to accurately forecast specific samples. Although researchers in the subsequent study [30] attempted to boost accuracy by employing the RBF neural network, which is known to be a fast-learner and accurate network, they failed to adequately address the issue of feature selection, which led to an excessive number of inputs being fed into the network. However, in the present study, an attempt was made to both improve accuracy and contribute useful inputs by probing and finding the gaps in prior studies. Seven features were proposed as useful in predicting job burnout using the GMDH neural network and its self-organization capabilities. This allowed for the accurate classification of all available samples. It would seem logical to choose a model based on how well it performed on the test set. It would lead to too optimistic generalization performance estimations to repeatedly use the test set. We will emphasize K-Fold cross-validation for model assessment in this research. The most popular method for model assessment and model selection in machine learning is K-fold cross-validation. K-Fold cross-validation’s key tenet is that every sample in our dataset gets a chance to be evaluated. It is a unique instance of cross-validation in which k times are iterated across a dataset collection. We divide the dataset into k parts for each round, using one part for validation and merging the remaining k-1 parts to create a training subset for model assessment. Since it is evident from Fig 5 that the error dispersion of the created neural networks against the test dataset is extremely low and the designed networks have a very high accuracy in identifying job burnout, we estimated K to be 20 and separated the data into training and testing data in 20 distinct modes. It should be noted that the selected variables in each fold were the same. Academics and professionals in the domains of business, entrepreneurship, organizational behavior, engineering sciences, and environmental problems will be interested in the outcomes of this study. due to a fall in the use of technology approaches in social science and humanities research, including neural network design. Managers and owners of many companies and start-ups can use the results of the current research to determine the job burnout of their employees and the employees they want to hire. Examining this important parameter will surely help in increasing the productivity of a company Investigating different individual characteristics and its effect on job burnout as well as using different neural networks in determining this parameter can be researchable topics for researchers in this field. Although the artificial neural network in this research correctly determined the relationship between inputs and output, it is very difficult to determine human behavior. One of the limitations of this research is the small number used small dataset. In future research, researchers can help the generalization and development of this research by examining more statistical communities using the methodology presented in this research. Considering that, the relationship between input and output is non-linear, expressing this relationship is very difficult and is considered as one of the limitations of this research. However, the presented neural network can be used to create a connection between the input and output data. In this case, when describing the relationships is very complicated and difficult, explainability methods such as SHAP can be used [35].

## 5. Conclusions

With the help of the GMDH neural network, a new numerical model for detecting burnout was developed in this research. Furthermore, the effective characteristics in identifying burnout have been identified utilizing this network. job experience, age, the stress of Covid-19, cynicism, exhaustion, professional efficiency, gender, resilience, married status, and having children were thought of as input factors, whereas burnout was thought of as an outcome. Quantitative methods were used in the examinations. A substantial fraction of the entrepreneurial community-384 directors and representatives from startups-were surveyed for this study. Information was gathered using a variety of tools, including the BRCS, the MBI-GS, and specialized measures of stress related to the Coronavirus. Cronbach’s alpha and an expert panel both affirmed the reliability of the polls. Considering that the GMDH neural network selects effective inputs in a self-organizing process, after the implementation of GMDH neural network, it was found that the characteristics of gender, marital status, job experience, professional efficiency, age, stress of covid-19, and resilience were selected as effective characteristics for predicting job burnout. The required information was collected via questionnaires and then split into two categories: training and test data. Through trial and error, we found the most error-free ANN architecture. The presented model makes use of 7 input features, 1 output, and 3 hidden layers comprised of 4, 3, and 2 neurons. The proposed neural network successfully categorized all data sets provided to it. The obtained results confirmed the validity of the presented model.

## Supporting information

S1 File (DOCX)
