# Correction: Introducing effective parameters for predicting job burnout using a self-organizing method based on group method of data handling neural network

**DOI:** 10.1371/journal.pone.0324283

**Published:** 2025-05-12

**Authors:** Tingting Fan, Ehsan Nazemi

There is an error in affiliation 2 for author Ehsan Nazemi. The correct affiliation 2 is: Independent researcher, Southampton, United Kingdom.
