# Supplementary material for: Introducing effective parameters for predicting job burnout using a self-organizing method based on group method of data handling neural network
Source: PLoS One. 2023 Nov 6;18(11):e0290267. doi: 10.1371/journal.pone.0290267 (PMC10627462; doi:10.1371/journal.pone.0290267)
Supplement: S1 File — (DOCX) [file pone.0290267.s001.docx]

**Introducing effective parameters for predicting job burnout using a self-organizing method based on group method of data handling neural network**

Tingting Fan ^1^, Ehsan Nazemi ^2,*^

^1^ Assumption University, Bangkok 10240, Thailand; [tingting6941@zjkju.edu.cn](mailto:tingting6941@zjkju.edu.cn)

^2^ Faculty of Engineering and Physical Sciences, Southampton University, UK e.nazemi@soton.ac.uk

**Supplementary data**

The input and output data used in this study to train the artificial neural network, are shown in the below table. The input data consists of seven qualitative factors (number of years in the employment, age, exposure to Covid-19 stress, level of weariness, level of cynicism, level of professional efficiency, and level of resilience) and three nominal variables (married status, gender, and having children).

| **Input** | | | | | | | | | | **Output** |
| --- | --- | --- | --- | --- | --- | --- | --- | --- | --- | --- |
| **age** | **gender** | **married status** | **number of years in the employment** | **having children** | **exposure to Covid-19 stress** | **exposure to Covid-19 stress** | **level of cynicism** | **level of professional efficiency** | **level of resilience** | **Burnout** |
| 35 | 0 | 0 | 6 | 0 | 7.45 | 3.28 | 4.89 | 3.12 | 17 | 2 |
| 42 | 0 | 1 | 13 | 1 | 8.9 | 5.23 | 2.13 | 4.85 | 11 | 1 |
| 44 | 0 | 1 | 15 | 1 | 7.61 | 4.44 | 3.11 | 4.15 | 10 | 1 |
| 39 | 1 | 1 | 11 | 1 | 9.8 | 6 | 2.13 | 5.54 | 8 | 1 |
| 29 | 1 | 0 | 3 | 0 | 6.54 | 1.2 | 5.56 | 2.16 | 18 | 2 |
| 40 | 0 | 0 | 15 | 0 | 1 | 2.3 | 6 | 3.1 | 16 | 2 |
| 26 | 0 | 0 | 1 | 0 | 3.33 | 1.1 | 5.55 | 1.8 | 19 | 2 |
| 46 | 0 | 1 | 18 | 1 | 7.88 | 5.47 | 2.24 | 3.49 | 12 | 1 |
| 40 | 1 | 1 | 11 | 1 | 10 | 6 | 0.3 | 4.98 | 9 | 1 |
| 35 | 1 | 1 | 4 | 0 | 7.76 | 3.4 | 4 | 2.63 | 19 | 2 |
| 41 | 1 | 0 | 12 | 0 | 8.89 | 3.5 | 3 | 2.9 | 8 | 1 |
| 52 | 0 | 1 | 18 | 1 | 6.66 | 5.03 | 2.01 | 6 | 9 | 1 |
| 50 | 0 | 1 | 13 | 1 | 10 | 5.87 | 0.5 | 5.41 | 11 | 1 |
| 38 | 0 | 0 | 6 | 0 | 1.9 | 0.4 | 4.68 | 0.4 | 19 | 2 |
| 37 | 1 | 1 | 8 | 1 | 10 | 6 | 1 | 6 | 5 | 1 |
| 49 | 0 | 1 | 26 | 1 | 6.4 | 6 | 2.08 | 5.43 | 11 | 1 |
| 53 | 0 | 1 | 24 | 1 | 8.84 | 4.9 | 0.35 | 5.11 | 13 | 1 |
| 44 | 1 | 1 | 16 | 1 | 10 | 6 | 1 | 6 | 6 | 1 |
| 36 | 1 | 1 | 7 | 0 | 6.43 | 4.33 | 2 | 5.63 | 15 | 1 |
| 38 | 1 | 1 | 8 | 1 | 9 | 4.01 | 3.45 | 3.98 | 14 | 1 |
| 40 | 0 | 0 | 11 | 0 | 4.32 | 2 | 3 | 2 | 18 | 2 |
| 39 | 1 | 1 | 5 | 1 | 9.87 | 6 | 1.3 | 6 | 7 | 1 |
| 33 | 1 | 1 | 4 | 1 | 5.5 | 2.1 | 4.32 | 2.1 | 17 | 2 |
| 40 | 0 | 1 | 11 | 1 | 7 | 4.5 | 3 | 3.9 | 14 | 1 |
| 55 | 0 | 1 | 27 | 1 | 5.5 | 5 | 2 | 5 | 8 | 1 |
| 29 | 1 | 0 | 2 | 0 | 6 | 2.1 | 4 | 2.1 | 18 | 2 |
| 35 | 0 | 1 | 3 | 0 | 3 | 0.33 | 6 | 0.33 | 18 | 2 |
| 38 | 0 | 0 | 6 | 0 | 5 | 0.33 | 6 | 0.33 | 19 | 2 |
| 44 | 0 | 1 | 15 | 1 | 8 | 6 | 0.28 | 6 | 5 | 1 |
| 48 | 1 | 1 | 19 | 1 | 10 | 5.5 | 1.2 | 5.4 | 6 | 1 |
| 60 | 0 | 1 | 29 | 1 | 7.7 | 4.89 | 3.2 | 6 | 4 | 1 |
| 24 | 0 | 0 | 1 | 0 | 5 | 0 | 6 | 0 | 18 | 2 |
| 57 | 1 | 1 | 28 | 1 | 10 | 5.6 | 0.4 | 6 | 9 | 1 |
| 46 | 1 | 1 | 18 | 0 | 6 | 4.3 | 6 | 2.3 | 12 | 1 |
| 37 | 0 | 0 | 9 | 0 | 4.3 | 3.5 | 3.8 | 0.3 | 19 | 2 |
| 34 | 0 | 0 | 7 | 0 | 7.77 | 3.42 | 4.4 | 3 | 16 | 2 |
| 41 | 0 | 1 | 14 | 1 | 9 | 5.5 | 2 | 4.9 | 5 | 1 |
| 44 | 0 | 1 | 16 | 1 | 8 | 4 | 3.1 | 4.5 | 18 | 2 |
| 39 | 1 | 1 | 10 | 1 | 10 | 6 | 2.22 | 5 | 9 | 1 |
| 35 | 1 | 0 | 5 | 0 | 5 | 1.4 | 5.66 | 0.3 | 19 | 2 |
| 42 | 0 | 0 | 16 | 0 | 2 | 2.9 | 5.9 | 3 | 17 | 2 |
| 27 | 0 | 0 | 3 | 0 | 3.5 | 1 | 6 | 0.2 | 16 | 2 |
| 49 | 0 | 1 | 19 | 1 | 8 | 5.9 | 2.3 | 3.56 | 12 | 1 |
| 41 | 1 | 1 | 10 | 1 | 9 | 6 | 0.2 | 5 | 13 | 1 |
| 35 | 1 | 1 | 6 | 0 | 8 | 3.1 | 4 | 1.2 | 20 | 2 |
| 40 | 1 | 0 | 11 | 0 | 8.9 | 3.6 | 3.2 | 1.2 | 11 | 1 |
| 50 | 0 | 1 | 19 | 1 | 6.66 | 5.6 | 1.03 | 6 | 9 | 1 |
| 52 | 0 | 1 | 20 | 1 | 9.9 | 6 | 0.6 | 5.41 | 10 | 1 |
| 40 | 0 | 0 | 17 | 0 | 2 | 0.2 | 4.6 | 0.3 | 20 | 2 |
| 39 | 1 | 1 | 7 | 0 | 10 | 5.5 | 0.6 | 6 | 6 | 1 |
| 50 | 0 | 1 | 29 | 1 | 6.6 | 5.9 | 2 | 5.63 | 4 | 1 |
| 55 | 0 | 1 | 27 | 1 | 8.9 | 5 | 0.35 | 5.29 | 7 | 1 |
| 48 | 1 | 1 | 18 | 1 | 9 | 6 | 1.2 | 6 | 9 | 1 |
| 37 | 1 | 1 | 8 | 0 | 5.6 | 4.35 | 1 | 5.93 | 16 | 2 |
| 36 | 1 | 1 | 4 | 1 | 10 | 4 | 3.3 | 6 | 10 | 1 |
| 45 | 0 | 0 | 10 | 0 | 4.52 | 1 | 3 | 2 | 19 | 2 |
| 42 | 1 | 1 | 9 | 1 | 10 | 6 | 1.3 | 5.5 | 7 | 1 |
| 30 | 1 | 1 | 2 | 0 | 5.5 | 1.2 | 4.32 | 2 | 17 | 2 |
| 42 | 0 | 1 | 10 | 1 | 7.7 | 4.6 | 3.01 | 3.9 | 12 | 1 |
| 58 | 0 | 1 | 32 | 1 | 5 | 5.3 | 2.03 | 5 | 13 | 1 |
| 30 | 1 | 0 | 5 | 1 | 6.3 | 2.2 | 4.05 | 2.1 | 20 | 2 |
| 29 | 0 | 1 | 1 | 0 | 3.2 | 0.2 | 5.6 | 0.3 | 17 | 2 |
| 38 | 0 | 0 | 5 | 0 | 5.5 | 1 | 6 | 0.8 | 18 | 2 |
| 45 | 0 | 1 | 16 | 1 | 9 | 5.7 | 0.3 | 5.3 | 10 | 1 |
| 60 | 1 | 1 | 33 | 1 | 9.8 | 6 | 1 | 5.4 | 7 | 1 |
| 49 | 0 | 1 | 25 | 1 | 7.6 | 4.3 | 3.3 | 6 | 4 | 1 |
| 25 | 0 | 0 | 1 | 0 | 5.5 | 1 | 6 | 1 | 19 | 2 |
| 58 | 1 | 1 | 29 | 1 | 9 | 6 | 0.4 | 6 | 8 | 1 |
| 44 | 1 | 1 | 19 | 1 | 6.3 | 2.3 | 5 | 5.1 | 9 | 1 |
| 39 | 0 | 0 | 7 | 0 | 4.4 | 3.6 | 3.5 | 0.1 | 17 | 2 |
| 36 | 0 | 0 | 5 | 0 | 7.11 | 3.77 | 4 | 3.64 | 18 | 2 |
| 41 | 1 | 1 | 14 | 1 | 8.36 | 5.1 | 2 | 4 | 15 | 1 |
| 45 | 0 | 1 | 16 | 1 | 7 | 4 | 3.14 | 4 | 14 | 1 |
| 40 | 1 | 1 | 12 | 1 | 10 | 6 | 2.12 | 5.63 | 8 | 1 |
| 28 | 1 | 0 | 4 | 0 | 6 | 2.3 | 5 | 2 | 18 | 2 |
| 41 | 0 | 0 | 16 | 0 | 2 | 2.2 | 6 | 3 | 20 | 2 |
| 29 | 0 | 0 | 5 | 0 | 3 | 1 | 6 | 1.5 | 20 | 2 |
| 48 | 0 | 1 | 19 | 1 | 7.04 | 5.5 | 2.2 | 3.36 | 12 | 1 |
| 39 | 1 | 1 | 12 | 1 | 9 | 5 | 0.2 | 4.96 | 11 | 1 |
| 33 | 1 | 1 | 5 | 0 | 7 | 3.7 | 4.33 | 2 | 18 | 2 |
| 40 | 1 | 0 | 13 | 0 | 8 | 3.1 | 3.55 | 0.45 | 12 | 1 |
| 53 | 0 | 1 | 19 | 1 | 6.32 | 5.6 | 2 | 6 | 9 | 1 |
| 55 | 0 | 1 | 14 | 1 | 9 | 5.5 | 0.6 | 5 | 10 | 1 |
| 39 | 0 | 0 | 9 | 0 | 1 | 0.14 | 4 | 0.8 | 19 | 2 |
| 38 | 1 | 1 | 10 | 0 | 8.86 | 5 | 1.3 | 5.91 | 5 | 1 |
| 47 | 0 | 1 | 24 | 1 | 10 | 6 | 2 | 5.33 | 11 | 1 |
| 53 | 0 | 1 | 25 | 1 | 10 | 5 | 0.7 | 5.13 | 13 | 1 |
| 48 | 1 | 1 | 20 | 1 | 9 | 5.5 | 1.22 | 5.79 | 6 | 1 |
| 35 | 1 | 1 | 15 | 0 | 6.51 | 4.1 | 2.01 | 4.59 | 15 | 1 |
| 40 | 1 | 1 | 10 | 1 | 10 | 3.5 | 3.79 | 3.61 | 14 | 1 |
| 41 | 0 | 0 | 12 | 0 | 4.62 | 2.3 | 3.54 | 2.33 | 8 | 1 |
| 38 | 1 | 1 | 9 | 1 | 9.87 | 6 | 1.22 | 5.55 | 7 | 1 |
| 34 | 1 | 1 | 6 | 1 | 5.36 | 2.2 | 4 | 2.7 | 20 | 2 |
| 41 | 0 | 1 | 12 | 1 | 7.01 | 4.4 | 2.98 | 4 | 13 | 1 |
| 59 | 0 | 1 | 28 | 1 | 5.4 | 5.41 | 2.1 | 6.3 | 16 | 1 |
| 30 | 1 | 0 | 3 | 0 | 6 | 2 | 4.6 | 2 | 18 | 2 |
| 33 | 0 | 1 | 4 | 0 | 3.2 | 0.7 | 5.4 | 0.4 | 18 | 2 |
| 39 | 0 | 0 | 7 | 0 | 5 | 0.2 | 5 | 0 | 19 | 2 |
| 45 | 0 | 1 | 15 | 1 | 10 | 5.85 | 0.2 | 5.8 | 4 | 1 |
| 50 | 1 | 1 | 20 | 1 | 9 | 5.1 | 3.33 | 6 | 5 | 1 |
| 59 | 0 | 1 | 30 | 1 | 10 | 4.3 | 3 | 5 | 6 | 1 |
| 25 | 0 | 0 | 2 | 0 | 6 | 0 | 6 | 1 | 18 | 2 |
| 58 | 1 | 1 | 26 | 1 | 9 | 6 | 0.1 | 5.23 | 7 | 1 |
| 49 | 1 | 1 | 18 | 0 | 6.32 | 4.01 | 6 | 2.3 | 10 | 1 |
| 38 | 0 | 0 | 10 | 0 | 5 | 3.8 | 4.4 | 0.3 | 20 | 2 |
| 35 | 0 | 0 | 7 | 0 | 7.8 | 3.7 | 4.3 | 4.3 | 19 | 2 |
| 40 | 0 | 1 | 15 | 1 | 7.96 | 6 | 2.45 | 6 | 6 | 1 |
| 45 | 0 | 1 | 17 | 0 | 8.63 | 6 | 3.66 | 6 | 18 | 2 |
| 40 | 1 | 1 | 11 | 1 | 9 | 5.36 | 2 | 5.1 | 9 | 1 |
| 36 | 1 | 0 | 6 | 0 | 4.44 | 1.39 | 6 | 0.2 | 19 | 2 |
| 41 | 0 | 0 | 15 | 0 | 2.1 | 2.89 | 6 | 4.4 | 17 | 2 |
| 28 | 0 | 0 | 3 | 0 | 3.66 | 1.4 | 6 | 0.6 | 15 | 2 |
| 50 | 0 | 1 | 20 | 1 | 8.44 | 5.1 | 2 | 3 | 12 | 1 |
| 42 | 1 | 1 | 11 | 1 | 10 | 5.41 | 0.3 | 6 | 11 | 1 |
| 36 | 1 | 1 | 9 | 0 | 9.63 | 3 | 4.6 | 1 | 18 | 2 |
| 41 | 1 | 0 | 13 | 0 | 9 | 3 | 3 | 1.89 | 10 | 1 |
| 52 | 0 | 1 | 20 | 1 | 7 | 6 | 1 | 4.35 | 9 | 1 |
| 50 | 0 | 1 | 22 | 1 | 10 | 5 | 0.9 | 5.67 | 10 | 1 |
| 41 | 0 | 0 | 19 | 0 | 3 | 0.1 | 5 | 0.4 | 19 | 2 |
| 38 | 1 | 1 | 7 | 0 | 9 | 6 | 0.5 | 5.25 | 6 | 1 |
| 51 | 0 | 1 | 30 | 1 | 7 | 6 | 0.3 | 5.19 | 6 | 1 |
| 54 | 0 | 1 | 26 | 1 | 10 | 6 | 0.2 | 5 | 13 | 1 |
| 49 | 1 | 1 | 20 | 1 | 10 | 5.77 | 1 | 5 | 7 | 1 |
| 39 | 1 | 1 | 9 | 1 | 5.4 | 4.23 | 2 | 6 | 16 | 2 |
| 35 | 1 | 1 | 5 | 1 | 9 | 3 | 3.5 | 6 | 4 | 1 |
| 44 | 0 | 0 | 11 | 0 | 4.6 | 1.4 | 4 | 0.3 | 17 | 2 |
| 41 | 1 | 1 | 10 | 1 | 10 | 6 | 1 | 5 | 7 | 1 |
| 31 | 1 | 1 | 3 | 0 | 5 | 1.4 | 5.5 | 2.3 | 16 | 2 |
| 43 | 0 | 1 | 11 | 1 | 7.8 | 4.6 | 3 | 4.53 | 12 | 1 |
| 56 | 0 | 1 | 31 | 1 | 6 | 5.6 | 2.4 | 4.63 | 10 | 1 |
| 32 | 1 | 0 | 6 | 1 | 6.7 | 0.2 | 5.4 | 0.8 | 19 | 2 |
| 30 | 0 | 1 | 2 | 0 | 3 | 1 | 6 | 1 | 17 | 2 |
| 36 | 0 | 0 | 6 | 0 | 5 | 1.3 | 6 | 1 | 19 | 2 |
| 44 | 0 | 1 | 17 | 1 | 10 | 6 | 0.4 | 6 | 10 | 1 |
| 57 | 1 | 1 | 31 | 1 | 10 | 5.55 | 1.2 | 6 | 7 | 1 |
| 50 | 0 | 1 | 29 | 1 | 7.8 | 4 | 3.4 | 5 | 4 | 1 |
| 28 | 0 | 0 | 6 | 0 | 5 | 1.2 | 5.5 | 1.3 | 20 | 2 |
| 59 | 1 | 1 | 28 | 1 | 10 | 5.54 | 0.2 | 6 | 8 | 1 |
| 45 | 1 | 1 | 20 | 1 | 6.5 | 3 | 6 | 5 | 13 | 1 |
| 38 | 0 | 0 | 8 | 0 | 4 | 3.7 | 3.3 | 0.5 | 17 | 2 |
| 36 | 0 | 0 | 7 | 0 | 7.77 | 3.1 | 4.5 | 3.33 | 16 | 2 |
| 41 | 0 | 1 | 14 | 0 | 6.89 | 5 | 2 | 4.16 | 14 | 1 |
| 47 | 0 | 1 | 13 | 1 | 7 | 4.9 | 3 | 4 | 13 | 1 |
| 40 | 1 | 1 | 19 | 1 | 10 | 6 | 2 | 5.78 | 8 | 1 |
| 30 | 1 | 0 | 4 | 0 | 6 | 2 | 6 | 2 | 18 | 2 |
| 41 | 0 | 0 | 19 | 0 | 1.5 | 2.2 | 5 | 3 | 19 | 2 |
| 29 | 0 | 0 | 2 | 0 | 3 | 1 | 5.55 | 1.5 | 20 | 2 |
| 45 | 0 | 1 | 19 | 1 | 7.11 | 6 | 2.24 | 4 | 12 | 1 |
| 41 | 1 | 1 | 12 | 1 | 9 | 5.54 | 0.4 | 5 | 7 | 1 |
| 33 | 1 | 1 | 5 | 0 | 7.63 | 3.4 | 3 | 3.3 | 18 | 2 |
| 40 | 1 | 0 | 13 | 0 | 8 | 3.6 | 4 | 2 | 14 | 1 |
| 51 | 0 | 1 | 19 | 1 | 6 | 5.02 | 2.7 | 5.12 | 13 | 1 |
| 52 | 0 | 1 | 14 | 1 | 10 | 6 | 0.6 | 5 | 10 | 1 |
| 39 | 0 | 0 | 8 | 0 | 1.78 | 1 | 4 | 0.9 | 19 | 2 |
| 36 | 1 | 1 | 9 | 1 | 9.6 | 5.3 | 1.5 | 4.45 | 5 | 1 |
| 50 | 0 | 1 | 27 | 1 | 6.11 | 4.5 | 2 | 6 | 5 | 1 |
| 54 | 0 | 1 | 25 | 1 | 10 | 6 | 0.34 | 5 | 4 | 1 |
| 45 | 1 | 1 | 17 | 1 | 9 | 5.58 | 1.05 | 5.36 | 4 | 1 |
| 37 | 1 | 1 | 8 | 0 | 6.88 | 4.5 | 2.41 | 5.93 | 9 | 1 |
| 39 | 1 | 1 | 9 | 1 | 9.47 | 4.22 | 3.77 | 3 | 14 | 1 |
| 41 | 0 | 0 | 12 | 0 | 4.61 | 2.3 | 3.2 | 2.06 | 15 | 1 |
| 40 | 1 | 1 | 12 | 1 | 10 | 5.44 | 1 | 5 | 6 | 1 |
| 35 | 1 | 1 | 5 | 1 | 5 | 2 | 4.7 | 2.79 | 17 | 2 |
| 41 | 0 | 1 | 12 | 1 | 8 | 4.7 | 2 | 3.44 | 14 | 1 |
| 56 | 0 | 1 | 28 | 1 | 5 | 5.4 | 3 | 6 | 16 | 1 |
| 30 | 1 | 0 | 4 | 0 | 6.7 | 2 | 6 | 2 | 18 | 2 |
| 33 | 0 | 1 | 2 | 0 | 3.4 | 1 | 5 | 0.4 | 19 | 2 |
| 39 | 0 | 0 | 8 | 0 | 5.6 | 0.2 | 5 | 2.1 | 20 | 2 |
| 42 | 0 | 1 | 13 | 1 | 8.9 | 6 | 1 | 6 | 9 | 1 |
| 49 | 1 | 1 | 19 | 1 | 8.41 | 5 | 1.8 | 5.4 | 6 | 1 |
| 59 | 0 | 1 | 30 | 1 | 7.36 | 1.4 | 3.5 | 5.5 | 4 | 1 |
| 25 | 0 | 0 | 2 | 0 | 5.6 | 0 | 6 | 1 | 18 | 2 |
| 60 | 1 | 1 | 27 | 1 | 9.5 | 5.7 | 0.2 | 5.6 | 9 | 1 |
| 45 | 1 | 1 | 19 | 0 | 5 | 4.4 | 5.5 | 2.4 | 10 | 1 |
| 38 | 0 | 0 | 10 | 0 | 4 | 3.4 | 3 | 0.8 | 20 | 2 |
| 35 | 0 | 0 | 8 | 0 | 7.3 | 3.1 | 4 | 3.4 | 16 | 2 |
| 40 | 0 | 1 | 15 | 1 | 10 | 6 | 3.2 | 4.63 | 6 | 1 |
| 42 | 0 | 1 | 17 | 1 | 10 | 6 | 3.5 | 4.17 | 18 | 2 |
| 40 | 1 | 1 | 11 | 1 | 9 | 5.5 | 2 | 5.16 | 9 | 1 |
| 36 | 1 | 0 | 6 | 0 | 5.6 | 1.3 | 6 | 0.4 | 19 | 2 |
| 41 | 0 | 0 | 13 | 0 | 2.4 | 2.8 | 6 | 3.3 | 20 | 2 |
| 29 | 0 | 0 | 2 | 0 | 3 | 1.4 | 5.59 | 1.9 | 16 | 2 |
| 50 | 0 | 1 | 18 | 1 | 9 | 6 | 2.1 | 3.4 | 17 | 2 |
| 42 | 1 | 1 | 11 | 1 | 10 | 5.59 | 0.3 | 5.63 | 18 | 2 |
| 37 | 1 | 1 | 7 | 0 | 8.3 | 3 | 4.5 | 1.03 | 18 | 2 |
| 41 | 1 | 0 | 12 | 0 | 8.4 | 3.5 | 3 | 1 | 13 | 1 |
| 51 | 0 | 1 | 20 | 1 | 6 | 6 | 1 | 5.45 | 9 | 1 |
| 53 | 0 | 1 | 21 | 1 | 9.03 | 5 | 0.6 | 5.71 | 12 | 1 |
| 41 | 0 | 0 | 18 | 0 | 2.7 | 0.2 | 4 | 0.2 | 19 | 2 |
| 38 | 1 | 1 | 9 | 0 | 10 | 6 | 0.35 | 6 | 6 | 1 |
| 52 | 0 | 1 | 30 | 1 | 6.1 | 6 | 0.32 | 5 | 12 | 1 |
| 54 | 0 | 1 | 26 | 1 | 8.9 | 6 | 1 | 6 | 13 | 1 |
| 49 | 1 | 1 | 20 | 1 | 9.6 | 5.56 | 1 | 6 | 7 | 1 |
| 38 | 1 | 1 | 9 | 0 | 5.4 | 4.11 | 1 | 6 | 15 | 1 |
| 37 | 1 | 1 | 5 | 1 | 9 | 4.63 | 3 | 5.59 | 17 | 2 |
| 44 | 0 | 0 | 11 | 0 | 7 | 1.33 | 3.1 | 2.33 | 17 | 2 |
| 40 | 1 | 1 | 12 | 1 | 10 | 5.56 | 1.4 | 6 | 7 | 1 |
| 31 | 1 | 1 | 6 | 0 | 6 | 1.04 | 4.21 | 2.3 | 17 | 2 |
| 43 | 0 | 1 | 12 | 1 | 7 | 4 | 3 | 4 | 12 | 1 |
| 59 | 0 | 1 | 31 | 1 | 4.5 | 6 | 2.7 | 5.45 | 15 | 1 |
| 32 | 1 | 0 | 6 | 1 | 5.3 | 1 | 4.11 | 2.36 | 19 | 2 |
| 31 | 0 | 1 | 2 | 0 | 3 | 1.2 | 6 | 0.4 | 17 | 2 |
| 39 | 0 | 0 | 6 | 0 | 5 | 1.3 | 5.4 | 0.7 | 18 | 2 |
| 44 | 0 | 1 | 17 | 1 | 10 | 6 | 0.4 | 6 | 10 | 1 |
| 59 | 1 | 1 | 32 | 1 | 10 | 5.66 | 1.1 | 6 | 7 | 1 |
| 50 | 0 | 1 | 26 | 1 | 8 | 4.65 | 3.4 | 5.33 | 4 | 1 |
| 27 | 0 | 0 | 2 | 0 | 5.6 | 1.04 | 6 | 1 | 19 | 2 |
| 59 | 1 | 1 | 30 | 1 | 10 | 5.59 | 0.2 | 5 | 8 | 1 |
| 45 | 1 | 1 | 20 | 1 | 6 | 2.01 | 6 | 0.4 | 15 | 1 |
| 40 | 0 | 0 | 9 | 0 | 4.5 | 3.71 | 3.6 | 0.5 | 17 | 2 |
| 36 | 0 | 0 | 7 | 0 | 8 | 3.33 | 4 | 3.33 | 16 | 2 |
| 41 | 0 | 1 | 17 | 1 | 8.09 | 5.23 | 2.88 | 6 | 5 | 1 |
| 45 | 0 | 1 | 13 | 1 | 7.63 | 6 | 3.77 | 6 | 5 | 1 |
| 38 | 1 | 1 | 10 | 1 | 10 | 5.44 | 2 | 6 | 8 | 1 |
| 30 | 1 | 0 | 1 | 0 | 6.4 | 3.47 | 5.1 | 2.44 | 18 | 2 |
| 41 | 0 | 0 | 14 | 0 | 1 | 3.21 | 5.95 | 3 | 17 | 2 |
| 31 | 0 | 0 | 8 | 0 | 2 | 1.02 | 6 | 1.6 | 15 | 2 |
| 45 | 0 | 1 | 20 | 1 | 10 | 6 | 0.5 | 3.51 | 13 | 1 |
| 39 | 1 | 1 | 12 | 1 | 9 | 5.56 | 0.2 | 4.96 | 13 | 1 |
| 36 | 1 | 1 | 6 | 0 | 7 | 3.3 | 4.63 | 2.03 | 18 | 2 |
| 40 | 1 | 0 | 13 | 0 | 8 | 3.7 | 3.33 | 2 | 4 | 1 |
| 51 | 0 | 1 | 19 | 1 | 6 | 6 | 2 | 4.56 | 5 | 1 |
| 49 | 0 | 1 | 14 | 1 | 9 | 6 | 0.8 | 5.66 | 9 | 1 |
| 39 | 0 | 0 | 7 | 0 | 1 | 0.1 | 4 | 0.7 | 20 | 2 |
| 41 | 1 | 1 | 9 | 1 | 10 | 5.31 | 1.6 | 5.9 | 6 | 1 |
| 50 | 0 | 1 | 27 | 1 | 6.1 | 5.55 | 2 | 5.12 | 5 | 1 |
| 52 | 0 | 1 | 25 | 1 | 8.6 | 4.9 | 0.6 | 6 | 13 | 1 |
| 43 | 1 | 1 | 14 | 1 | 9 | 5.13 | 1.4 | 5.35 | 9 | 1 |
| 37 | 1 | 1 | 10 | 0 | 6 | 4.01 | 2.6 | 5.49 | 10 | 1 |
| 39 | 1 | 1 | 9 | 1 | 8 | 4 | 3.47 | 3.61 | 14 | 1 |
| 41 | 0 | 0 | 12 | 0 | 4 | 2.03 | 3.22 | 2.37 | 19 | 2 |
| 42 | 1 | 1 | 6 | 1 | 10 | 5.11 | 1.01 | 5.81 | 6 | 1 |
| 32 | 1 | 1 | 5 | 1 | 5.22 | 2 | 4 | 2 | 15 | 2 |
| 40 | 0 | 1 | 12 | 1 | 8.32 | 4.2 | 3.33 | 4 | 12 | 1 |
| 53 | 0 | 1 | 28 | 1 | 4.9 | 6 | 2 | 6 | 11 | 1 |
| 30 | 1 | 0 | 7 | 0 | 5 | 0.3 | 6 | 2 | 20 | 2 |
| 36 | 0 | 1 | 4 | 0 | 2 | 1 | 5.12 | 0.8 | 19 | 2 |
| 40 | 0 | 0 | 7 | 0 | 3 | 0.6 | 5.89 | 0.6 | 18 | 2 |
| 45 | 0 | 1 | 16 | 1 | 7 | 5.45 | 0.5 | 5.3 | 9 | 1 |
| 49 | 1 | 1 | 19 | 1 | 9 | 5.21 | 1.7 | 6 | 6 | 1 |
| 59 | 0 | 1 | 30 | 1 | 7.9 | 4.9 | 3.4 | 5.1 | 4 | 1 |
| 26 | 0 | 0 | 2 | 0 | 6 | 0 | 5.51 | 0 | 18 | 2 |
| 56 | 1 | 1 | 29 | 1 | 9 | 5.7 | 0.3 | 5.5 | 9 | 1 |
| 45 | 1 | 1 | 19 | 0 | 6.33 | 4.02 | 5.55 | 2.6 | 13 | 1 |
| 38 | 0 | 0 | 10 | 0 | 3.33 | 3.4 | 3.9 | 0.33 | 19 | 2 |
| 35 | 0 | 0 | 8 | 0 | 8 | 3.5 | 4 | 3.1 | 16 | 2 |
| 40 | 0 | 1 | 15 | 1 | 10 | 6 | 2.77 | 4.8 | 6 | 1 |
| 46 | 0 | 1 | 17 | 1 | 10 | 6 | 3.4 | 4.5 | 18 | 2 |
| 41 | 1 | 1 | 13 | 1 | 10 | 5.54 | 2.11 | 5 | 9 | 1 |
| 36 | 1 | 0 | 6 | 0 | 6 | 1 | 6 | 0.2 | 19 | 2 |
| 42 | 0 | 0 | 17 | 0 | 1 | 1 | 6 | 3.5 | 18 | 2 |
| 29 | 0 | 0 | 4 | 0 | 4 | 1.66 | 6 | 0.3 | 19 | 2 |
| 50 | 0 | 1 | 20 | 1 | 8.21 | 6 | 2 | 4.6 | 12 | 1 |
| 40 | 1 | 1 | 11 | 1 | 9.41 | 5.88 | 0.9 | 6 | 8 | 1 |
| 36 | 1 | 1 | 7 | 0 | 8.3 | 3.4 | 4.6 | 1.5 | 18 | 2 |
| 41 | 1 | 0 | 10 | 0 | 6.4 | 3.4 | 3 | 1.3 | 14 | 1 |
| 52 | 0 | 1 | 20 | 1 | 8 | 6 | 1 | 5.54 | 9 | 1 |
| 50 | 0 | 1 | 21 | 1 | 10 | 5.89 | 0.7 | 5.31 | 10 | 1 |
| 43 | 0 | 0 | 18 | 0 | 3 | 0.3 | 4.4 | 0.2 | 19 | 2 |
| 40 | 1 | 1 | 8 | 0 | 9.5 | 5.3 | 0.5 | 5.46 | 6 | 1 |
| 52 | 0 | 1 | 30 | 1 | 7 | 6 | 0.9 | 5.6 | 12 | 1 |
| 53 | 0 | 1 | 28 | 1 | 9 | 6 | 0.7 | 5.3 | 13 | 1 |
| 49 | 1 | 1 | 19 | 1 | 10 | 5.59 | 1.4 | 6 | 7 | 1 |
| 38 | 1 | 1 | 9 | 0 | 6 | 4.51 | 1.5 | 6 | 20 | 2 |
| 38 | 1 | 1 | 5 | 1 | 9 | 4.3 | 3 | 5 | 14 | 1 |
| 44 | 1 | 0 | 11 | 0 | 4.7 | 0.4 | 3.2 | 3.3 | 16 | 2 |
| 43 | 1 | 1 | 10 | 1 | 10 | 5.7 | 1 | 6 | 7 | 1 |
| 30 | 1 | 1 | 3 | 0 | 5 | 1.1 | 4.1 | 1.2 | 20 | 2 |
| 42 | 0 | 1 | 11 | 1 | 7.9 | 5.3 | 3 | 4 | 10 | 1 |
| 58 | 0 | 1 | 24 | 1 | 5.6 | 6 | 2 | 5.6 | 9 | 1 |
| 30 | 1 | 0 | 9 | 1 | 6.1 | 0.3 | 4.3 | 3 | 19 | 2 |
| 29 | 0 | 1 | 3 | 0 | 3.6 | 1 | 6 | 0.2 | 17 | 2 |
| 38 | 0 | 0 | 6 | 0 | 5 | 0.2 | 5.54 | 0.9 | 18 | 2 |
| 45 | 0 | 1 | 17 | 1 | 9.6 | 5.9 | 0.4 | 5.5 | 10 | 1 |
| 60 | 1 | 1 | 30 | 1 | 10 | 4.1 | 1.7 | 6 | 7 | 1 |
| 49 | 0 | 1 | 24 | 1 | 7.7 | 6 | 3.11 | 5 | 5 | 1 |
| 25 | 0 | 0 | 3 | 0 | 5.6 | 1 | 6 | 1.3 | 19 | 2 |
| 58 | 1 | 1 | 30 | 1 | 10 | 5.3 | 0.2 | 5 | 9 | 1 |
| 44 | 1 | 1 | 20 | 1 | 7 | 2 | 5.57 | 6 | 15 | 1 |
| 39 | 0 | 0 | 8 | 0 | 5.2 | 3.7 | 3.6 | 0 | 17 | 2 |
| 35 | 0 | 0 | 7 | 0 | 8 | 3.7 | 4.15 | 3.14 | 16 | 2 |
| 42 | 0 | 1 | 14 | 1 | 9 | 5.5 | 2 | 4.99 | 18 | 2 |
| 44 | 0 | 1 | 16 | 1 | 8.3 | 4.4 | 3.01 | 4.66 | 16 | 1 |
| 39 | 1 | 1 | 12 | 1 | 10 | 5.3 | 2 | 5.78 | 8 | 1 |
| 29 | 1 | 0 | 4 | 0 | 7.6 | 1.4 | 6 | 2.11 | 18 | 2 |
| 40 | 0 | 0 | 16 | 0 | 1.2 | 2.4 | 5.78 | 3 | 17 | 2 |
| 26 | 0 | 0 | 2 | 0 | 3.6 | 1 | 5.4 | 1.9 | 15 | 2 |
| 46 | 0 | 1 | 19 | 1 | 7.84 | 5.3 | 2.04 | 5.3 | 4 | 1 |
| 40 | 1 | 1 | 12 | 1 | 9 | 5.4 | 0.12 | 5 | 5 | 1 |
| 35 | 1 | 1 | 5 | 0 | 7.8 | 4 | 0.9 | 2 | 18 | 2 |
| 41 | 1 | 0 | 13 | 0 | 10 | 4 | 3.3 | 3 | 14 | 1 |
| 52 | 0 | 1 | 19 | 1 | 6.9 | 5.5 | 2 | 5.36 | 8 | 1 |
| 50 | 0 | 1 | 14 | 1 | 10 | 6 | 0.2 | 4.66 | 10 | 1 |
| 38 | 0 | 0 | 7 | 0 | 1 | 0.2 | 4.5 | 0.2 | 19 | 2 |
| 37 | 1 | 1 | 10 | 1 | 9 | 5.4 | 0.2 | 5.43 | 4 | 1 |
| 49 | 0 | 1 | 27 | 1 | 8 | 6.3 | 0.3 | 5.21 | 12 | 1 |
| 53 | 0 | 1 | 25 | 1 | 9 | 5 | 1 | 5.12 | 13 | 1 |
| 44 | 1 | 1 | 17 | 1 | 9 | 5.1 | 1.4 | 6 | 6 | 1 |
| 36 | 1 | 1 | 8 | 0 | 6.3 | 4.3 | 2.01 | 5.41 | 15 | 1 |
| 38 | 1 | 1 | 9 | 1 | 10 | 5 | 3.8 | 3.5 | 14 | 1 |
| 40 | 0 | 0 | 13 | 0 | 4.5 | 2.1 | 3.66 | 3 | 17 | 2 |
| 39 | 1 | 1 | 6 | 1 | 10 | 4.9 | 1.5 | 5 | 6 | 1 |
| 33 | 1 | 1 | 5 | 1 | 6.1 | 2.4 | 4.77 | 2 | 20 | 2 |
| 40 | 0 | 1 | 12 | 1 | 7.8 | 5 | 3.1 | 4 | 14 | 1 |
| 55 | 0 | 1 | 29 | 1 | 5 | 5.3 | 2.01 | 6 | 16 | 1 |
| 29 | 1 | 0 | 3 | 0 | 6.3 | 1 | 6 | 2 | 18 | 2 |
| 35 | 0 | 1 | 2 | 0 | 2 | 0.7 | 5.11 | 0.2 | 17 | 2 |
| 38 | 0 | 0 | 7 | 0 | 6 | 0.2 | 5.4 | 0.2 | 18 | 2 |
| 44 | 0 | 1 | 16 | 1 | 10 | 5.9 | 0.9 | 5.93 | 9 | 1 |
| 48 | 1 | 1 | 20 | 1 | 9 | 6 | 1 | 6 | 6 | 1 |
| 59 | 0 | 1 | 27 | 1 | 7.8 | 4.6 | 3.4 | 5.36 | 4 | 1 |
| 24 | 0 | 0 | 1 | 0 | 6 | 1.02 | 5.5 | 1 | 18 | 2 |
| 56 | 1 | 1 | 27 | 1 | 9 | 6 | 0.3 | 6 | 9 | 1 |
| 45 | 1 | 1 | 19 | 0 | 5.4 | 4.5 | 5.58 | 2 | 5 | 1 |
| 38 | 0 | 0 | 10 | 0 | 3.6 | 3.2 | 3.47 | 0.2 | 19 | 2 |
| 35 | 0 | 0 | 8 | 0 | 8 | 3.7 | 4 | 3.65 | 18 | 2 |
| 40 | 0 | 1 | 13 | 1 | 10 | 4 | 2.14 | 5 | 6 | 1 |
| 43 | 0 | 1 | 15 | 1 | 10 | 5.5 | 3.77 | 4.5 | 18 | 2 |
| 37 | 1 | 1 | 11 | 1 | 9 | 5.9 | 2 | 5.77 | 9 | 1 |
| 34 | 1 | 0 | 6 | 0 | 6 | 1.6 | 6 | 0.2 | 19 | 2 |
| 40 | 0 | 0 | 17 | 0 | 1 | 3 | 6 | 3.2 | 17 | 2 |
| 29 | 0 | 0 | 1 | 0 | 4.2 | 1.7 | 5.54 | 0.6 | 20 | 2 |
| 50 | 0 | 1 | 20 | 1 | 10 | 6 | 2 | 3.4 | 7 | 1 |
| 41 | 1 | 1 | 11 | 1 | 9.5 | 5.4 | 0.1 | 6 | 9 | 1 |
| 35 | 1 | 1 | 7 | 0 | 8.5 | 3.4 | 4.35 | 1 | 18 | 2 |
| 41 | 1 | 0 | 12 | 0 | 8.4 | 3.5 | 3.3 | 1 | 14 | 1 |
| 51 | 0 | 1 | 20 | 1 | 7 | 5.9 | 1.04 | 5.3 | 9 | 1 |
| 52 | 0 | 1 | 21 | 1 | 10 | 6 | 0.5 | 5.22 | 10 | 1 |
| 43 | 0 | 0 | 18 | 0 | 1 | 0.3 | 4.63 | 0.5 | 19 | 2 |
| 42 | 1 | 1 | 8 | 0 | 3 | 5.1 | 0.8 | 5.41 | 6 | 1 |
| 51 | 0 | 1 | 30 | 1 | 7 | 6 | 2.1 | 6 | 12 | 1 |
| 54 | 0 | 1 | 28 | 1 | 9 | 6 | 0.2 | 5.93 | 13 | 1 |
| 49 | 1 | 1 | 16 | 1 | 10 | 5.1 | 1 | 5 | 7 | 1 |
| 36 | 1 | 1 | 5 | 0 | 4.2 | 4.3 | 0.2 | 6 | 15 | 2 |
| 37 | 1 | 1 | 5 | 1 | 10 | 5 | 3.4 | 6 | 14 | 1 |
| 44 | 0 | 0 | 11 | 0 | 5 | 1.3 | 3.5 | 2.31 | 16 | 2 |
| 43 | 1 | 1 | 10 | 1 | 9 | 5.5 | 1.2 | 5 | 7 | 1 |
| 31 | 1 | 1 | 4 | 0 | 6.3 | 1.9 | 4.11 | 2.1 | 19 | 2 |
| 40 | 0 | 1 | 11 | 1 | 7 | 4.5 | 3 | 4 | 12 | 1 |
| 59 | 0 | 1 | 30 | 1 | 6.12 | 6 | 2.2 | 5.63 | 13 | 1 |
| 31 | 1 | 0 | 3 | 1 | 8 | 0.2 | 4 | 2 | 19 | 2 |
| 30 | 0 | 1 | 2 | 0 | 3.96 | 1 | 6 | 0.2 | 17 | 2 |
| 39 | 0 | 0 | 6 | 0 | 5 | 1.3 | 5.54 | 0.9 | 18 | 2 |
| 44 | 0 | 1 | 17 | 1 | 10 | 6 | 0.4 | 6 | 10 | 1 |
| 60 | 1 | 1 | 32 | 1 | 10 | 6 | 1.2 | 6 | 7 | 1 |
| 50 | 0 | 1 | 26 | 1 | 7.12 | 4.5 | 3.01 | 4.56 | 5 | 1 |
| 26 | 0 | 0 | 1 | 0 | 5.4 | 1.3 | 5.54 | 0.2 | 19 | 2 |
| 59 | 1 | 1 | 25 | 1 | 8.36 | 7 | 0.2 | 6 | 9 | 1 |
| 43 | 1 | 1 | 20 | 1 | 6.23 | 2 | 6 | 5 | 15 | 1 |
| 38 | 0 | 0 | 8 | 0 | 4.5 | 3.4 | 4 | 0 | 17 | 2 |
| 36 | 0 | 0 | 7 | 0 | 8 | 3.33 | 4 | 3.12 | 18 | 2 |
| 41 | 0 | 1 | 14 | 1 | 9 | 5.21 | 2.77 | 4.88 | 15 | 1 |
| 45 | 0 | 1 | 19 | 1 | 10 | 6 | 3.02 | 4.16 | 11 | 1 |
| 38 | 1 | 1 | 12 | 1 | 9 | 5.95 | 2 | 5 | 8 | 1 |
| 30 | 1 | 0 | 6 | 0 | 6.3 | 2.3 | 5.3 | 2.4 | 18 | 2 |
| 42 | 0 | 0 | 16 | 0 | 1.3 | 2.2 | 5.4 | 3 | 19 | 2 |
| 27 | 0 | 0 | 2 | 0 | 4 | 1 | 6 | 1.9 | 20 | 2 |
| 45 | 0 | 1 | 19 | 1 | 9 | 5.1 | 0.1 | 3.45 | 13 | 1 |
| 41 | 1 | 1 | 12 | 1 | 10 | 6 | 0.22 | 4.96 | 14 | 1 |
| 36 | 1 | 1 | 5 | 0 | 9 | 3.04 | 4.5 | 3.22 | 18 | 2 |
| 40 | 1 | 0 | 13 | 0 | 8 | 3.05 | 3.01 | 3 | 14 | 1 |
| 50 | 0 | 1 | 16 | 1 | 7 | 6 | 2 | 6 | 8 | 1 |
| 51 | 0 | 1 | 14 | 1 | 9 | 6 | 0.66 | 5.6 | 10 | 1 |
| 36 | 0 | 0 | 5 | 0 | 1 | 0.2 | 4 | 0.2 | 19 | 2 |
| 38 | 1 | 1 | 9 | 1 | 9 | 5.89 | 0 | 6.37 | 5 | 1 |
| 50 | 0 | 1 | 24 | 1 | 6.5 | 5.41 | 2 | 5 | 11 | 1 |
| 52 | 0 | 1 | 25 | 1 | 8.3 | 6 | 0.23 | 5.32 | 13 | 1 |
| 41 | 1 | 1 | 17 | 1 | 9 | 5.57 | 1.04 | 5 | 6 | 1 |
| 35 | 1 | 1 | 8 | 0 | 6 | 4.44 | 2.3 | 6 | 10 | 1 |
| 37 | 1 | 1 | 9 | 1 | 10 | 6 | 3.7 | 4 | 14 | 1 |
| 39 | 0 | 0 | 12 | 0 | 4.5 | 1 | 2.9 | 2.11 | 19 | 2 |
| 40 | 1 | 1 | 6 | 1 | 9 | 5.4 | 1.4 | 5 | 6 | 1 |
| 32 | 1 | 1 | 5 | 1 | 5 | 2 | 4 | 2.1 | 19 | 2 |
| 41 | 0 | 1 | 10 | 1 | 9 | 4.63 | 3.2 | 3.8 | 14 | 1 |
| 54 | 0 | 1 | 24 | 1 | 5 | 6 | 1.2 | 5.59 | 15 | 1 |
| 30 | 1 | 0 | 3 | 0 | 6.3 | 1 | 4.12 | 2.3 | 18 | 2 |
| 33 | 0 | 1 | 5 | 0 | 3.4 | 0.29 | 6 | 0.3 | 18 | 2 |
| 39 | 0 | 0 | 7 | 0 | 5 | 0.21 | 5.5 | 0 | 20 | 2 |
| 40 | 0 | 1 | 16 | 1 | 9 | 5.59 | 2 | 4.78 | 11 | 1 |
| 49 | 1 | 1 | 20 | 1 | 10 | 5.3 | 1 | 5 | 6 | 1 |
| 60 | 0 | 1 | 30 | 1 | 7.8 | 4.69 | 3.3 | 5.5 | 4 | 1 |
| 27 | 0 | 0 | 2 | 0 | 4.5 | 1 | 5.5 | 0.5 | 18 | 2 |
| 56 | 1 | 1 | 25 | 1 | 9 | 5.8 | 0 | 6 | 10 | 1 |
| 45 | 1 | 1 | 19 | 0 | 6.3 | 4.5 | 6 | 2 | 16 | 1 |
